# Contrast-Enhanced CT Texture Analysis in Colon Cancer: Correlation with Genetic Markers

**DOI:** 10.3390/tomography8050184

**Published:** 2022-08-31

**Authors:** Filippo Crimì, Chiara Zanon, Giulio Cabrelle, Kim Duyen Luong, Laura Albertoni, Quoc Riccardo Bao, Marta Borsetto, Elisa Baratella, Giulia Capelli, Gaya Spolverato, Matteo Fassan, Salvatore Pucciarelli, Emilio Quaia

**Affiliations:** 1 Institute of Radiology, Department of Medicine-DIMED, University of Padova, 35128 Padova, Italy; 2 Pathology and Cytopathology Unit, Department of Medicine, University of Padova, 35128 Padua, Italy; 3 General Surgery 3, Department of Surgical, Oncological, and Gastroenterological Sciences (DiSCOG), University of Padova, 35128 Padova, Italy; 4 Department of Radiology, Cattinara Hospital, University of Trieste, 34127 Trieste, Italy; 5 Veneto Institute of Oncology, IOV-IRCCS, 35128 Padua, Italy

**Keywords:** texture analysis, colorectal cancer, computed tomography, genetic markers, microsatellite instability, mismatch repair, KRAS, NRAS, BRAF

## Abstract

**Background:** The purpose of the study was to determine whether contrast-enhanced CT texture features relate to, and can predict, the presence of specific genetic mutations involved in CRC carcinogenesis. **Materials and methods:** This retrospective study analyzed the pre-operative CT in the venous phase of patients with CRC, who underwent testing for mutations in the KRAS, NRAS, BRAF, and MSI genes. Using a specific software based on CT images of each patient, for each slice including the tumor a region of interest was manually drawn along the margin, obtaining the volume of interest. A total of 56 texture parameters were extracted that were compared between the wild-type gene group and the mutated gene group. A *p*-value of <0.05 was considered statistically significant. **Results:** The study included 47 patients with stage III-IV CRC. Statistically significant differences between the MSS group and the MSI group were found in four parameters: GLRLM RLNU (area under the curve (AUC) 0.72, sensitivity (SE) 77.8%, specificity (SP) 65.8%), GLZLM SZHGE (AUC 0.79, SE 88.9%, SP 65.8%), GLZLM GLNU (AUC 0.74, SE 88.9%, SP 60.5%), and GLZLM ZLNU (AUC 0.77, SE 88.9%, SP 65.8%). **Conclusions**: The findings support the potential role of the CT texture analysis in detecting MSI in CRC based on pre-treatment CT scans.

## 1. Introduction

Colorectal cancer (CRC) is the third leading cause of cancer globally, which accounted for 9.4% of the overall cancer deaths in 2020 [1]. The mortality rate has decreased in many countries due to early detection and primary prevention efforts. However, survival in advanced colorectal cancer remains poor [2], since liver metastases occur in 15–25% of patients at diagnosis and are the leading cause of death [3]. 

CRC is a successful model for the development of genetic testing in oncology [4]. Currently, several genetic mutations used in clinical practice have been identified. Approximately 15% have microsatellite instability (MSI) and mismatch repair (MMR) protein deficiency [5]. The recent guidelines indicate the usefulness of the genetic characterization of CRC as a predictive marker for the response to chemotherapy and targeted therapies, and as a prognostic indicator of the patient outcome [6]. Currently, due to the reduced cost of genetic testing, the genotyping of tumor biomarkers such as BRAF, KRAS, NRAS, and MSI is possible [7]. Usually, mutations are diagnosed via the genetic testing of a tumor biopsy; however, this is an invasive and expensive procedure with possible sampling errors, and often it does not represent the vast heterogeneity of the tumor [8]. 

A texture analysis (TA) is a helpful technique designed to extract, from radiologic images, additional information that is not easily depicted via visual inspection [9]. Computed tomography (CT) associated with TA (CT-TA) can function as a “virtual biopsy” of indeterminate masses. This procedure involves mathematical calculations performed with the data contained within the images. CT-TA is an ongoing field of research, and it has shown promise in differentiating between benign and malignant lesions in different organs for various cancers [10]. Several studies have already shown that CT-TA correlates with the prognosis in lung cancer, head and neck cancer, metastatic renal cell carcinoma, colon cancer liver metastasis, and esophageal cancer [11]. Thus, the purpose of our study was to define whether contrast-enhanced CT-TA features may predict the presence of specific genetic mutations involved in CRC carcinogenesis.

## 2. Materials and Methods

### 2.1. Patient Selection 

This was a retrospective observational study approved by the local ethics committee (protocol N. 0035301, 23 May 2022). All patients with a pathological diagnosis of locally colorectal adenocarcinoma (stage III and IV according to the American Joint Committee on Cancer 8th Edition [12]) between January 2014 and December 2019 (*n* = 110) were extracted from the prospectively maintained database in our third-level university hospital. The inclusion criteria were also the presence of a portal venous-phase CT scan with a slice thickness ≤ 3 mm performed within a month before surgery and molecular testing for genetic mutations of the primary resected tumor, specifically the MSI, BRAF, NRAS, and KRAS. Patients without a contrast-enhanced CT scan or mutational status of the genes tested and with appendiceal cancer, an undiscernible tumor upon CT due to artifacts or stages I–II colon cancers [12] were excluded.

### 2.2. CT Image Acquisition 

All CT examinations were performed using a 64-slice scanner (Somatom Sensation, Siemens Healthineers, Erlangen, Germany). The intravenous contrast medium (2 mL/kg of Iohexol—Omnipaque™ 300 mgI/mL) was administered via the antecubital vein. The portal venous phase was acquired at 80 s after intravenous contrast media injection. The CT scan data were acquired using the following parameters: 120 kVp; 250 mA collimation 64 × 0.6 mm; rotation speed 0.5 s; pitch 0.8; slice thickness 3 mm. 

### 2.3. Texture Analysis 

**Figure 1:**
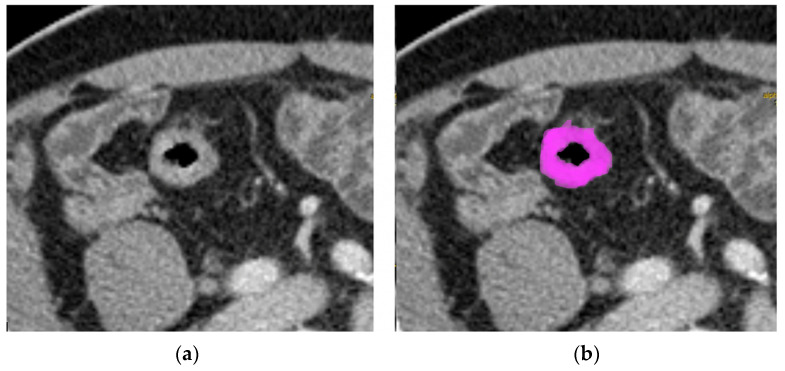
Tumor tissue in the axial CT portal venous phase images in locally advanced CRC in a 77-year-old man (**a**). The pink area represents the regions of interest; the procedure was applied for every single slice where the tumor was detectable, obtaining a 3D-ROI (**b**).

Each portal venous CT scan was retrieved from the institutional archive system, which were anonymized and loaded onto an independently developed open-access image analysis software program for the texture analysis (LIFEx—Local Image Features Extraction- Orsay, France) [13]. All CTs were resampled to a voxel size of 1 × 1 × 3 mm (X spacing, Y spacing, Z spacing). Two abdominal radiologists (15 and 5 years of experience), blinded to clinical outcomes and genetic profiles, identified the primary CRC, and a region of interest (ROI) was manually drawn along the tumor in the coronal, axial, and sagittal views, where the CRC appeared visible, excluding healthy tissue and the lumen of the intestinal segments. Particular attention was paid to exclude mucous and endoluminal materials and the healthy colic wall from ROI. Thus, a volume of interest (VOI) for each tumor was then obtained. LIFEx Software was used to analyze the voxels within the entire VOI and to compute a set of textural parameters for each VOI (Figure 1). 

### 2.4. Statistical Analysis 

According to the results of the mutational tests, the extracted parameters were divided to determine the association between the textural parameters and gene mutations. For each of the tested genes (i.e., KRAS, NRAS, BRAF, and microsatellite status), we split the patients into two groups: group 0 included patients with a wild-type (WT) gene or who were microsatellite-stable (MSS); group 1 included patients with genetic mutations or MSI. The normality of continuous variables was assessed using the Shapiro–Wilk test. In cases of abnormal distribution, the variables were described by the median and interquartile range (IQR). The texture parameters were compared between the two groups using the Mann–Whitney test and Bonferroni correction. A *p*-value of <0.05 was considered statistically significant. For significantly different texture parameters, a receiver operating characteristic (ROC) curve was plotted. The area under the curve (AUC) and the Youden index were extracted to determine the relationship between the CT-TA features and the genetic mutations.

## 3. Results

### 3.1. Patient Characteristics 

**Figure 2:**
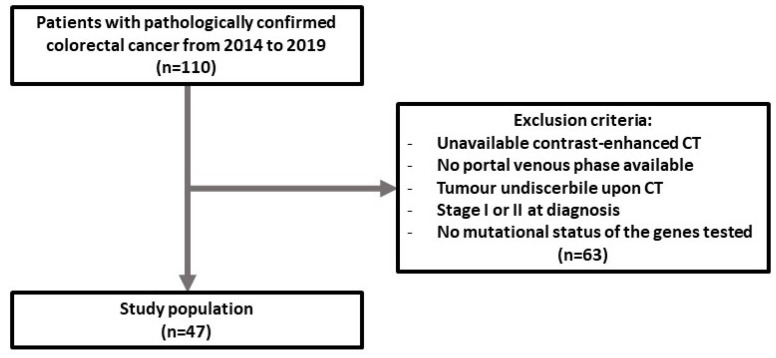
Flow chart of the enrolled population.

Forty-seven patients (27 men; median age: 70 years; IQR: 26–87) with locally advanced colorectal adenocarcinoma were included in the study (Figure 2). Each patient’s sex, age, genetic mutation status (BRAF, NRAS, KRAS), and MSI status based on tumor genetic profiling; location of the primary tumor; site of metastasis; and time from diagnosis to death were collected from the hospital information system. The population’s general characteristics, CRC stage and location, and genetic mutation distribution are reported in Table 1. 

### 3.2. Texture Values

**Figure 3:**
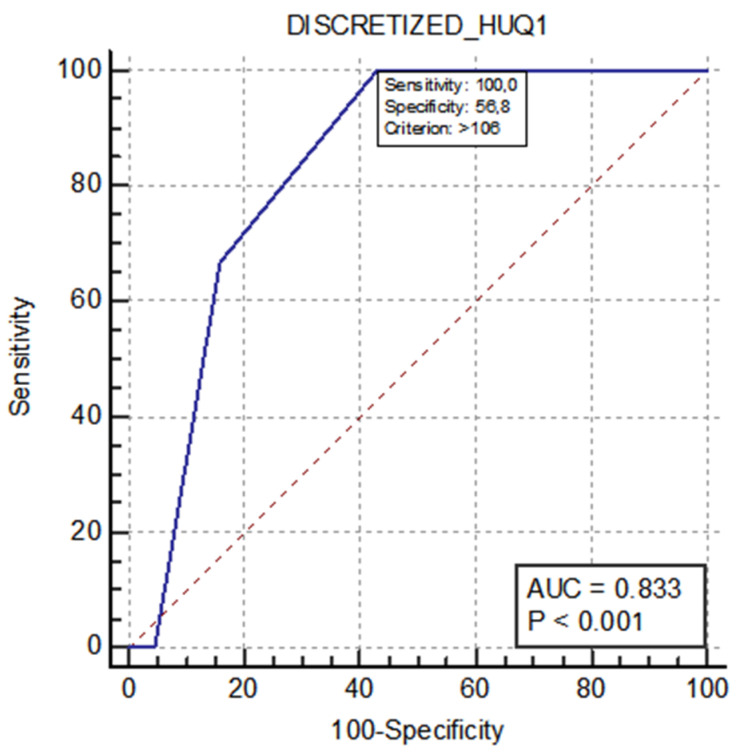
ROC curve for the prediction of NRAS mutations based on the discretized HU Q1.

**Figure 4:**
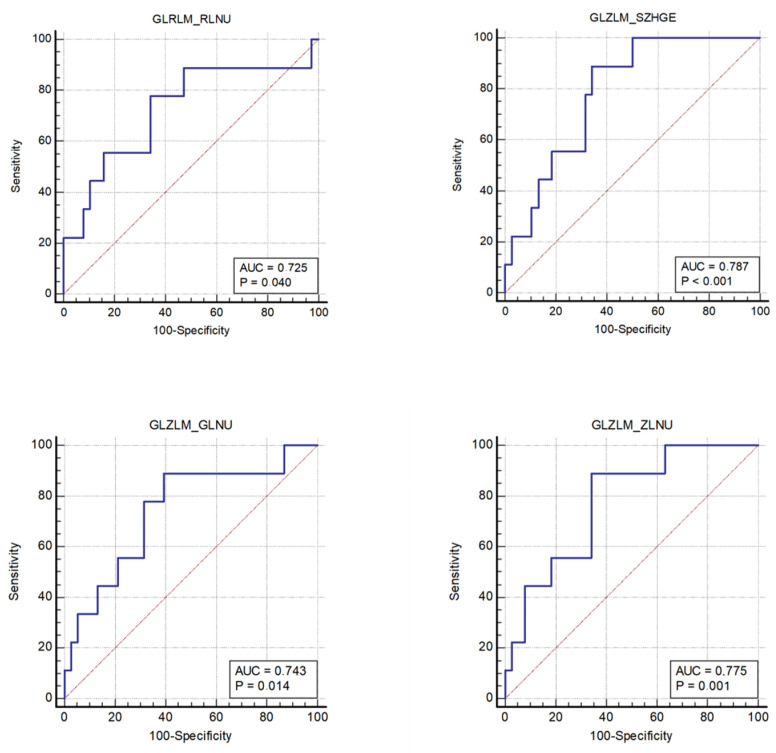
The microsatellite status with ROC curves of the 4 significant CT-TA parameters.

A total of 56 textural parameters were extracted, deriving from histogram, run length matrix (RLM), gray-level co-occurrence matrix (GLCM), gray-level run length matrix (GLRLM), and neighboring gray-level dependence matrix (NGLDM). For the KRAS and BRAF genes, none of the analyzed parameters showed a statistically significant difference between the two groups (Tables S1 and S2). A significant difference between the groups was found for NRAS for a textural parameter derived from the analysis of the attenuation values in the VOIs, the discretized Hounsfield Unit Quartile-1 (HU Q1) (Table 2 and Table S3). The ROC curve for prediction of the NRAS mutation based on the discretized HU Q1 is shown in Figure 3. For the MSS, 4 significant parameters were found between the two groups (Table 3):

- The nonuniformity of the lengths of the homogeneous runs (*GLRLM RLNU*), which was significantly higher in patients with MSI (AUC 0.725, sensitivity 77.8%, specificity 65.8%);
- The distribution of the short homogeneous zones with high gray levels (*GLZLM SZHGE*), which was significantly lower in patients with MSI (AUC 0.787, sensitivity 88.9%, specificity 65.8%);
- The nonuniformity of the gray levels (*GLZLM GLNU*), which was significantly higher in patients with MSI (AUC 0.743, sensitivity 88.9%, specificity 60.5%);
- The nonuniformity of the lengths of the homogeneous zones (*GLZLM ZLNU*), which was significantly higher in patients with MSI (AUC 0.775, sensitivity 88.9%, specificity 65.8%). For these parameters, the ROC curves were obtained (Figure 4, Table S4).

## 4. Discussion

The purpose of this study was to assess the correlation between contrast-enhanced CT-TA features and genetic aspects in CRC, in particular BRAF, NRAS, KRAS, and MSI.

In our study, the data obtained from the CT-TA in colorectal adenocarcinoma did not show any correlation with KRAS or BRAF gene mutations. Among the 56 examined textural parameters, only one (discretized *HU Q1*) showed a significant statistical difference between the NRAS wild-type group and the NRAS-mutated group. The discretized *HU Q1* represents the first quartile (Q1) of the ordinal scale of the discretized attenuation values (HU), and from the perspective of CT images, it identifies a low-attenuation region. The difference we found can be interpreted as that in the NRAS wild-type tumors, the darkest gray values would tend to be darker compared to those in the NRAS-mutated tumors. However, other related parameters (e.g., *HU Q2*, *HU Q3*) did not show any consistent results with this observation. Moreover, the NRAS-mutated group comprised only 3 patients, reflecting the sparse prevalence of NRAS-mutated colon cancers (3–5%) [14]. Thus, the TA parameter we found is not appealing in predicting the NRAS mutational status. 

The results support, on the other hand, the potential role of TAs in detecting MSI CRC based on pre-treatment portal venous CT scans. By comparing the MSS and MSI groups, 4 significant parameters emerged: *GLRLM RLNU* (which describes the nonuniformity of the lengths of the homogeneous runs), *GLZLM SZHGE* (which describes the distribution of the short homogeneous zones with high gray levels), *GLZLM GLNU* (which describes the nonuniformity of the gray levels), and *GLZLM ZLNU* (which describes the nonuniformity of the lengths of the homogeneous zones). These parameters wholly provide information for each gray level on the sizes of the homogeneous zones and on the sizes of the homogeneous runs. The values obtained are consistent and suggest there is less gray-level homogeneity in the images of MSI tumors compared to those of MSS tumors. The detection of the imaging heterogeneity in MSI colorectal adenocarcinoma could reflect the morphological heterogeneity of MSI tumors. Smedt et al., in a 60 patient study focusing on morphological tumor heterogeneity, found that this characteristic is a notable feature in MSI with respect to MSS CRC [15]. Histologically, MSI CRC is more inclined to display mixed morphological patterns such as glandular, mucinous, and solid aspects [16]. 

Previous studies have investigated the potential relationship between genetic mutations in CRC and radiological images, mainly using 18F-FDG PET/CT SUVmax, but with controversial results [17,18,19]. A meta-analysis showed the low sensitivity and specificity of 18F-FDG PET/CT in predicting the KRAS mutation in CRC [20], and more recently Taguchi et al. found an association between two CT-TA parameters (skewness and max value) and the KRAS mutational status [21]. The authors also showed that a model with 14 CT-TA parameters had superior prediction performance compared to the previously studied 18F-FDG PET/CT SUVmax. Similarly, other authors proposed a CT texture-based approach to predict the KRAS mutation [22,23]. Regarding the relationship between imaging features and the MSI status, Pernicka et al. showed that CT TA can predict the MSI status with low sensitivity (32%) and high specificity (95%) [24]. In an analogous study, Fan et al. found a sensitivity of 52% and a specificity of 86% [25]. In a study that involved dual-energy CT imaging, Wu et al. developed a model to predict MSI by integrating texture parameters and clinical features [26]. All of these authors concluded that the combined model of clinical variables and CT-TA imaging features was more effective in predicting MSI than the clinical or imaging features alone. In a recent study, Zhang et al. obtained similar results using an MRI texture analysis to predict MSI in rectal cancer [27]. 

Currently, the genetic status of colorectal adenocarcinoma is assessed via the histopathological examination of endoscopic or surgical resection samples. Biopsy remains the primary source of information for tumor classification and staging. However, tissue biopsies are prone to sampling errors principally deriving from lesion heterogeneity or the nondiagnostic sample size [28]. On the other hand, a TA provides an opportunity to study the entire lesion and multiple tumor sites. Moreover, TAs could better determine which tumor areas need to be biopsied according to the probability of mutations. Because WT and mutated CRCs have different treatment strategies, the potential role of CT-TA in predicting mutations could also help to plan individualized treatments without exposing patients to invasive procedures and without additional costs. 

Indeed, it has been demonstrated that for certain types of mutations, a targeted therapy is feasible (e.g., KRAS G12C inhibitor); hence, the knowledge of which mutations are present inside a colon cancer before surgery could be of paramount importance for the oncologists in order to set up a correct and targeted chemotherapy [29,30]. 

The current study points toward several future perspectives. The correlations between the analyzed TA parameters and genetic mutations should be thoroughly investigated in larger studies with representative populations to be validated or rejected conclusively, and an area for further investigation could also be a quantitative image analysis in arterial-phase and unenhanced CT scans. 

Our study has several limitations. First of all, the number of patients included in this analysis is not large enough to draw definitive conclusions, even though many previous studies on the CT-TA of colon cancer have investigated a similar number of cases. Additionally, the technique is time-consuming for the operator, who must accurately outline the tumor in all CT slices using special software, making the procedure difficult to reproduce. It has sometimes proved difficult to accurately delineate the contours of the tumor by excluding healthy tissue and the intestinal lumen. In the future, the use of automated segmentation and artificial intelligence may achieve the reproducibility of the technique. 

## 5. Conclusions

The CT-TA of colorectal adenocarcinoma has a potential role in predicting MSI. GLRLM RLNU, GLZLM SZHGE, GLZLM GLNU, GLZLM, and ZLNU showed significant different distributions between tumors with MSI and MSS. These results should be investigated in larger cohorts of patients to be validated. In the future, a validated radiological assessment of genetic mutations of CRC could decrease the time interval between the diagnosis and treatment, and could potentially act as a virtual biopsy.

## Figures and Tables

**Table 1:** Demographic and clinical–pathological characteristics of the studied population.

Characteristics	N (%)
**Sex**	
Males	27 (57%)
Females	20 (43%)
**Age (years)**	
Median	70
IQR	26–87
**Body mass index (kg/m^2^)**	
Median	24.6
IQR	19.1–31.8
**Stages**	
III	16 (33%)
IV	31 (66%)
**Tumor locations**	
Rectum-sigma	18 (38%)
Ascending colon	8 (17%)
Tranverse	8 (17%)
Descending	7 (15%)
Ciecum	6 (13%)
**Genetic mutations**	
BRAF	7 (15%)
KRAS	18 (38%)
NRAS	3 (6%)
MMR	9 (19%)

**Table 2:** Discretized *HU Q1* results. Note: IQR could not be calculated, as group 1 only comprised three patients; SE: sensitivity; SP: specificity.

Median (IQR)		*p*-Value	SE	SP	AUC (95% CI)	*p*
Group 0 NRAS	Group 1 NRAS					
106 (105–107)	108	0.049	100%	56.8%	0.833 (0.696–0.926)	<0.001

Legend: AUC: area under the curve; CI: confidence interval; SE: sensitivity; SP: specificity.

**Table 3:** Microsatellite status—significant parameters.

CT-TA Parameters	Group 0 (MSS) Median (IQR)	Group 1 (MSI) Median (IQR)	*p*	YI	SE (%)	SP (%)	AUC (95% CI)	*p*
GLRLM RLNU	4419 (2811–9267)	11829 (5918–21721)	0.037	0.44	77.8	65.8	0.725 (0.575–0.845)	0.040
GLZLM SZHGE	7334 (7114–7457)	7070 (6937–7192)	0.0081	0.55	88.9	65.8	0.787 (0.643–0.892)	0.001
GLZLM GLNU	97.39 (62.29–177.79)	186.42 (133.31–290.23)	0.025	0.49	88. 9	60.5	0.743 (0.594–0.859)	0.014
GLZLM ZLNU	378.96 (304.16–763.17)	920.71 (546.78–1378.71)	0.011	0.55	88.9	65.8	0.775 (0.630–0.884)	0.001

Legend: AUC: area under the curve; CI: confidence Interval; GLNU, gray-level nonuniformity; GLRLM, gray-level run length matrix; GLZLM: gray-level zone length matrix; RLNU: run length nonuniformity; SE: sensitivity; SP: specificity; SZHGE: short-zone high gray-level emphasis; YI: Youden Index; ZLNU: zone length nonuniformity.

## Data Availability

The data presented in this study are available on request from the corresponding author.

## Supplementary Materials

The following supporting information can be downloaded at: https://www.mdpi.com/article/10.3390/tomography8050184/s1, Table S1: KRAS gene: values of the 56 texture parameters. No significant parameters were found; Table S2: BRAF gene: values of the 56 texture parameters. No significant parameters were found; Table S3: NRAS gene: values of the 56 texture parameters. One significant parameter was found. Note: the interquartile range could not be calculated for the mutated NRAS group, as it only comprised three patients; Table S4: Microsatellite status: values of the 56 texture parameters. Four significant parameters were found.
