# Supplementary material for: Contrast-Enhanced CT Texture Analysis in Colon Cancer: Correlation with Genetic Markers
Source: Tomography. 2022 Aug 31;8(5):2193–201. doi: 10.3390/tomography8050184 (PMC9498512; doi:10.3390/tomography8050184)
Supplement: Supplementary file 1 [file tomography-08-00184-s001.zip › tomography-1865819-supplementary.pdf]

tomography MDPI

Supplementary Materials

Table S1. KRAS gene: values of the 56 texture parameters. No significant parameters were found.

Texture parameter	Median (interquartile range)		*p*
	Wild-type KRAS (group 0)	Mutated KRAS (group 1)	
**CONVENTIONAL HU ExcessKurtosis**	0.87 (0.33 to 1.39)	1.03 (0.51 to 1.56)	0.47
**CONVENTIONAL HU Kurtosis**	3.87 (3.33 to 4.39)	4.03 (3.51 to 4.56)	0.47
**CONVENTIONAL HU max**	159.74 (147.35 to 182.26)	167.4 (154.64 to 175.66)	0.68
**CONVENTIONAL HU mean**	74.78 (63.82 to 82.71)	73.43 (62.15 to 81.17)	0.76
**CONVENTIONAL HU min**	−60.28 (−85.99 to −41.77)	−87.4 (−107.56 to −39.83)	0.47
**CONVENTIONAL HU Q1**	59.56 (47.66 to 68.41)	59.9 (47.21 to 67.17)	0.95
**CONVENTIONAL HU Q2**	75.37 (65.37 to 85.14)	75.67 (64.6 to 83.95)	0.95
**CONVENTIONAL HU Q3**	89.73 (81.7 to 99.76)	89.44 (82.76 to 99.95)	0.84
**CONVENTIONAL HU Skewness**	−0.44 (−0.61 to −0.17)	−0.48 (−0.72 to −0.27)	0.42
**CONVENTIONAL HU std**	24.58 (22.6 to 27.09)	25 (23.05 to 28.36)	0.79
**DISCRETIZED AUC CSH**	1079.77 (1068.78 to 1087.67)	1078.45 (1067.17 to 1086.21)	0.74
**DISCRETIZED HISTO Energy [=Uniformity]**	0.12 (0.11 to 0.13)	0.12 (0.11 to 0.13)	0.84
**DISCRETIZED HISTO Entropy log2**	3.33 (3.19 to 3.47)	3.33 (3.22 to 3.48)	0.91
**DISCRETIZED HISTO Entropy log10**	1 (0.96 to 1.04)	1 (0.97 to 1.05)	0.91
**DISCRETIZED HU ExcessKurtosis**	0.85 (0.32 to 1.35)	1.02 (0.48 to 1.54)	0.44
**DISCRETIZED HU Kurtosis**	3.85 (3.32 to 4.35)	4.02 (3.48 to 4.54)	0.44
**DISCRETIZED HU max**	116 (115 to 119)	117 (116 to 118)	0.67
**DISCRETIZED HU mean**	107.98 (106.88 to 108.77)	107.84 (106.72 to 108.62)	0.74
**DISCRETIZED HU min**	94 (91.75 to 96.25)	91.5 (90 to 97)	0.47
**DISCRETIZED HU Q1**	106 (105 to 107)	106 (105 to 107)	0.95
**DISCRETIZED HU Q2**	108 (107 to 109)	108 (107 to 109)	0.92
**DISCRETIZED HU Q3**	109 (109 to 110)	109.5 (109 to 110)	0.83
**DISCRETIZED HU Skewness**	−0.44 (−0.6 to −0.16)	−0.47 (−0.7 to −0.26)	0.46
**DISCRETIZED HU std**	2.48 (2.28 to 2.72)	2.51 (2.32 to 2.85)	0.79
**GLCM Contrast [=Variance]**	6.57 (5.54 to 8)	6.64 (6.14 to 7.73)	0.74
**GLCM Correlation**	0.39 (0.34 to 0.44)	0.37 (0.34 to 0.47)	0.66
**GLCM Dissimilarity**	1.98 (1.79 to 2.17)	1.94 (1.91 to 2.13)	0.78
**GLCM Energy [=AngularSecondMoment]**	0.017 (0.014 to 0.021)	0.018 (0.015 to 0.021)	0.6
**GLCM Entropy log2 [=JointEntropy]**	6.4 (6.11 to 6.65)	6.34 (6.18 to 6.52)	0.95
**GLCM Entropy log10**	1.93 (1.84 to 2)	1.91 (1.86 to 1.96)	0.95
**GLCM Homogeneity [=InverseDifference]**	0.46 (0.44 to 0.48)	0.46 (0.44 to 0.47)	0.9
**GLRLM GLNU**	667.57 (474.06 to 1504.18)	1153.88 (327.3 to 1710.11)	0.73
**GLRLM HGRE**	1.16E+4 (1.14E+4 to 1.18E+4)	1.16E+4 (1.13E+4 to 1.17E+4)	0.74
**GLRLM LGRE**	8.6E-5 (8.5E-5 to 8.8E-5)	8.6E-5 (8.5E-5 to 8.8E-5)	0.68
**GLRLM LRE**	1.53 (1.48 to 1.59)	1.53 (1.46 to 1.55)	0.79
**GLRLM LRHGE**	1.76E+4 (1.72E+4 to 1.87E+4)	1.78E+4 (1.71E+4 to 1.81E+4)	0.78
**GLRLM LRLGE**	0.00013 (0.00013 to 0.00014)	0.00013 (0.00013 to 0.00014)	0.95
**GLRLM RLNU**	4661.98 (3227.46 to 11351.41)	6679.19 (2451.24 to 11364.2)	0.93
**GLRLM RP**	0.87 (0.85 to 0.88)	0.87 (0.86 to 0.88)	0.71

**GLRLM SRE**	0.9 (0.89 to 0.91)	0.9 (0.89 to 0.91)	0.63
**GLRLM SRHGE**	1.04E+4 (1.02E+4 to 1.06E+4)	1.05E+4 (1.02E+4 to 1.05E+4)	0.97
**GLRLM SRLGE**	7.7E-5 (7.6E-5 to 7.9E-5)	7.7E-5 (7.7E-5 to 8E-5)	0.69
**GLZLM GLNU**	104.25 (69.1 to 190.46)	132.52 (59.73 to 190.73)	0.81
**GLZLM HGZE**	1.14E+4 (1.12E+4 to 1.16E+45)	1.14E+4 (1.12E+4 to 1.16E+4)	0.42
**GLZLM LGZE**	8.8E-5 (8.6E-5 to 8.9E-5)	8.8E-5 (8.6E-5 to 9E-5)	0.42
**GLZLM LZE**	2573.42 (1176.92 to 6522.26)	3044.99 (1086.13 to 8368.48)	0.95
**GLZLM LZHGE**	3.07E+7 (1.42E+7 to 7.74E+7)	3.59E+07 (1.3E+07 to 9.72E+07)	0.98
**GLZLM LZLGE**	0.22 (0.098 to 0.55)	0.26 (0.09 to 0.72)	0.93
**GLZLM SZE**	0.63 (0.62 to 0.65)	0.65 (0.64 to 0.65)	0.17
**GLZLM SZHGE**	7232.64 (7009.95 to 7428.23)	7297.75 (7138.55 to 7441.65)	0.53
**GLZLM SZLGE**	5.6E-5 (5.4E-5 to 5.7E-5)	5.7E-5 (5.5E-5 to 5.9E-5)	0.12
**GLZLM ZLNU**	493.62 (328.08 to 950.03)	586.02 (316.99 to 897.54)	0.81
**GLZLM ZP**	0.16 (0.14 to 0.17)	0.16 (0.13 to 0.21)	0.41
**NGLDM Busyness**	0.34 (0.19 to 0.58)	0.42 (0.13 to 0.62)	1
**NGLDM Coarseness**	0.0012 (0.00048 to 0.0016)	0.00074 (0.00043 to 0.0022)	0.74
**NGLDM Contrast**	0.038 (0.025 to 0.047)	0.035 (0.028 to 0.043)	0.53

Legend: **AUC:** area under the curve; **CSH**: cumulative SUV-volume histogram**; GLCM**: gray-level-co-occurrence-matrix; **GLNU**, gray-level nonuniformity; **GLRLM**, gray-level-run-length-matrix; **GLZLM**: gray-level zone-length-matrix**; HGRE:** high-gray-level-emphasis**; HGZE**: high-gray-level-zone-emphasis; **HU:** Hounsfield-Unit**; LGZE:** low-gray-level-zone-emphasis; **LRE:** long-run-emphasis; **LRHGE:** long-run-high-gray-level-emphasis; **LRLGE:** long-run-low-gray-level-emphasis; **LZE**: large-zone-emphasis**; LZHGE**: large-zone-high-gray-level-emphasis; **; LZLGE**: large-zone-low-gray-level-emphasis; **NGLDM**: neighboring-gray-level-dependence-matrix; **Q1-2-3**: quartile 1-2-3; **RLNU**: run-length-non-uniformity**; RP:** Run-percentage; **SRHGE**: short-run-high-gray-level-emphasis**; SRLGE**: short-run-low-gray-level-emphasis; **SZHGE**: small-zone-high-gray-level-emphasis; **SZLGE**: small-zone-low-gray-level-emphasis**; SRE**: short-run-emphasis; **Std:** standard deviation; **SZE**: small-zone-emphasis; **SZHGE**: short-zone-high-gray-level-emphasis; ; **SZLGE**: short-zone-low-gray-level emphasis; **YI**: Youden Index; **ZLNU:** zone-length-non-uniformity; **ZP**: zone-percentage.

Table S2. BRAF gene: values of the 56 texture parameters. No significant parameters were found.

Texture parameter	Median (interquartile range)		p
	Wild-type BRAF (group 0)	Mutated BRAF (group 1)	
**CONVENTIONAL HU ExcessKurtosis**	0.93 (0.49 to 1.58)	1.01 (0.029 to 1.26)	0.63
**CONVENTIONAL HU Kurtosis**	3.93 (3.49 to 4.58)	4.01 (3.03 to 4.26)	0.63
**CONVENTIONAL HU max**	165.38 (151.49 to 178.04)	152.67 (143.15 to 176.87)	0.32
**CONVENTIONAL HU mean**	74.73 (63.71 to 81.83)	71.6 (64.25 to 80.79)	0.79
**CONVENTIONAL HU min**	−66.45 (−97.89 to −41.17)	−44.73 (−108.24 to −35.37)	0.70
**CONVENTIONAL HU Q1**	59.9 (47.51 to 68.43)	57.98 (48.08 to 62.71)	0.61
**CONVENTIONAL HU Q2**	75.98 (65.23 to 84.51)	73.45 (64.88 to 82.96)	0.77
**CONVENTIONAL HU Q3**	91.92 (82.42 to 99.82)	87.55 (81.09 to 100.46)	0.79
**CONVENTIONAL HU Skewness**	−0.44 (−0.66 to −0.2)	−0.52 (−0.62 to −0.28)	0.74
**CONVENTIONAL HU std**	24.73 (22.88 to 27.37)	24.53 (22.51 to 28.71)	0.90
**DISCRETIZED AUC CSH**	1079.78 (1068.69 to 1086.82)	1076.6 (1069.22 to 1085.76)	0.79
**DISCRETIZED HISTO Energy [=Uniformity]**	0.12 (0.11 to 0.13)	0.12 (0.099 to 0.13)	0.74
**DISCRETIZED HISTO Entropy log2**	3.34 (3.2 to 3.47)	3.33 (3.21 to 3.56)	0.88
**DISCRETIZED HISTO Entropy log10**	1 (0.96 to 1.05)	1 (0.97 to 1.07)	0.88
**DISCRETIZED HU ExcessKurtosis**	0.88 (0.46 to 1.52)	0.98 (0.027 to 1.25)	0.65
**DISCRETIZED HU Kurtosis**	3.88 (3.46 to 4.52)	3.98 (3.03 to 4.25)	0.65
**DISCRETIZED HU max**	117 (115.5 to 118.5)	116 (115 to 118.5)	0.60
**DISCRETIZED HU mean**	107.98 (106.87 to 108.68)	107.66 (106.92 to 108.58)	0.79
**DISCRETIZED HU min**	94 (91 to 96.5)	96 (89.25 to 96.75)	0.74
**DISCRETIZED HU Q1**	106 (105 to 107)	106 (105.25 to 107)	0.84
**DISCRETIZED HU Q2**	108 (107 to 109)	108 (107 to 108.75)	0.70
**DISCRETIZED HU Q3**	110 (109 to 110)	109 (109 to 110.5)	0.64
**DISCRETIZED HU Skewness**	−0.43 (−0.65 to −0.18)	−0.51 (−0.61 to −0.27)	0.72
**DISCRETIZED HU std**	2.49 (2.31 to 2.75)	2.47 (2.27 to 2.89)	0.93
**GLCM Contrast [=Variance]**	6.59 (5.79 to 8.02)	6.7 (5.72 to 7.37)	0.98
**GLCM Correlation**	0.38 (0.34 to 0.44)	0.41 (0.35 to 0.44)	0.44
**GLCM Dissimilarity**	1.94 (1.83 to 2.16)	1.99 (1.82 to 2.1)	0.98
**GLCM Energy [=AngularSecondMoment]**	0.017 (0.015 to 0.021)	0.017 (0.012 to 0.02)	0.55
**GLCM Entropy log2 [=JointEntropy]**	6.39 (6.14 to 6.57)	6.36 (6.18 to 6.79)	0.70
**GLCM Entropy log10**	1.92 (1.85 to 1.98)	1.91 (1.86 to 2.05)	0.70
**GLCM Homogeneity [=InverseDifference]**	0.46 (0.44 to 0.48)	0.45 (0.44 to 0.48)	0.81
**GLRLM GLNU**	804.89 (470.88 to 1567.22)	1400.92 (578.25 to 2265.58)	0.61
**GLRLM HGRE**	11650 (11418 to 11805)	11585 (11433 to 11789)	0.81
**GLRLM LGRE**	0.000086 (0.000085 to 0.000088)	0.000087 (0.000085 to 0.000088)	0.81
**GLRLM LRE**	1.53 (1.46 to 1.58)	1.54 (1.5 to 1.59)	0.88
**GLRLM LRHGE**	17842 (17175 to 18465)	17647 (17603 to 18492)	0.93
**GLRLM LRLGE**	1.3E-4 (1.3E-4 to 1.4E-4)	1.3E-4 (1.3E-4 to 1.4E-4)	0.57

**GLRLM RLNU**	5005 (2926 to 10445)	7525 (3662 to 15047)	0.59
**GLRLM RP**	0.87 (0.86 to 0.88)	0.86 (0.85 to 0.87)	0.74
**GLRLM SRE**	0.9 (0.89 to 0.91)	0.9 (0.89 to 0.9)	0.65
**GLRLM SRHGE**	10491 (10255 to 10620)	10318 (10252 to 10515)	0.42
**GLRLM SRLGE**	7.7E-5 (7.6E-5 to 7.9E-5)	7.7E-5 (7.7E-5 to 7.8E-5)	0.90
**GLZLM GLNU**	111.09 (62.84 to 188.52)	140.38 (74.81 to 236.44)	0.63
**GLZLM HGZE**	11436 (11272 to 11657)	11367 (11243 to 11640)	0.74
**GLZLM LGZE**	8.8E-5 (8.6E-5 to 8.9E-5)	8.8E-5 (8.6E-5 to 8.9E-5)	0.74
**GLZLM LZE**	2496 (1056 to 6682)	3486 (2045 to 11522)	0.51
**GLZLM LZHGE**	2.99E+7 (1.25E+7 to 7.88E+7)	4.22E+7 (2.38E+7 to 1.36E+7)	0.53
**GLZLM LZLGE**	0.21 (0.090 to 0.57)	0.29 (0.18 to 0.97)	0.47
**GLZLM SZE**	0.64 (0.62 to 0.65)	0.62 (0.62 to 0.65)	0.49
**GLZLM SZHGE**	7297 (7101 to 7449)	7179 (6947 to 7318)	0.14
**GLZLM SZLGE**	5.6E-5 (5.5E-5 to 5.8E-5)	5.5E-5 (5.3E-5 to 5.9E-5)	0.51
**GLZLM ZLNU**	495.79 (313.37 to 845)	547.07 (344.8 to 1067.06)	0.51
**GLZLM ZP**	0.16 (0.14 to 0.2)	0.15 (0.12 to 0.16)	0.34
**NGLDM Busyness**	0.36 (0.19 to 0.58)	0.51 (0.26 to 0.78)	0.46
**NGLDM Coarseness**	0.00095 (0.00048 to 0.0017)	0.0006 (0.00036 to 0.0013)	0.63
**NGLDM Contrast**	0.036 (0.026 to 0.043)	0.04 (0.027 to 0.047)	0.44

Legend: **AUC:** area under the curve; **CSH**: cumulative SUV-volume histogram**; GLCM**: gray-level-co-occurrence-matrix; **GLNU**, gray-level nonuniformity; **GLRLM**, gray-level-run-length-matrix; **GLZLM**: gray-level zone-length-matrix**; HGRE:** high-gray-level-emphasis**; HGZE**: high-gray-level-zone-emphasis; **HU:** Hounsfield-Unit**; LGZE:** low-gray-level-zone-emphasis; **LRE:** long-run-emphasis; **LRHGE:** long-run-high-gray-level-emphasis; **LRLGE:** long-run-low-gray-level-emphasis; **LZE**: large-zone-emphasis**; LZHGE**: large-zone-high-gray-level-emphasis; **; LZLGE**: large-zone-low-gray-level-emphasis; **NGLDM**: neighboring-gray-level-dependence-matrix; **Q1-2-3**: quartile 1-2-3; **RLNU**: run-length-non-uniformity**; RP:** Run-percentage; **SRHGE**: short-run-high-gray-level-emphasis**; SRLGE**: short-run-low-gray-level-emphasis; **SZHGE**: small-zone-high-gray-level-emphasis; **SZLGE**: small-zone-low-gray-level-emphasis**; SRE**: short-run-emphasis; **Std:** standard deviation; **SZE**: small-zone-emphasis; **SZHGE**: short-zone-high-gray-level-emphasis; ; **SZLGE**: short-zone-low-gray-level emphasis; **YI**: Youden Index; **ZLNU:** zone-length-non-uniformity; **ZP**: zone-percentage.

Table S3. NRAS gene: values of the 56 texture parameters. One significant parameter was found. Note: the interquartile range could not be calculated for the mutated NRAS group, as it only comprised three patients.

Texture parameter	Median (interquartile range)		*p*
	Wild-type NRAS (group 0)	Mutated NRAS (group 1)	
**CONVENTIONAL HU min**	−69.15 (−103.6 to −41.17)	−42.93 (NC)	0.19
**CONVENTIONAL HU mean**	72.43 (63.62 to 81.40)	86.60 (NC)	0.09
**CONVENTIONAL HU std**	24.63 (22.88 to 27.75)	24.19 (NC)	0.49
**CONVENTIONAL HU max**	164.79 (150.71 to 178.04)	149.59 (NC)	0.73
**CONVENTIONAL HU Q1**	59.57 (47.19 to 66.15)	73.55 (NC)	0.08
**CONVENTIONAL HU Q2**	73.83 (64.77 to 83.32)	88.46 (NC)	0.07
**CONVENTIONAL HU Q3**	87.34 (81.95 to 98.78)	100.82 (NC)	0.08
**CONVENTIONAL HU Skewness**	−0.43 (−0.59 to −0.18)	−0.69 (NC)	0.07
**CONVENTIONAL HU Kurtosis**	3.90 (3.44 to 4.32)	4.60 (NC)	0.21
**CONVENTIONAL HU ExcessKurtosis**	0.90 (0.44 to 1.32)	1.60 (NC)	0.21
**DISCRETIZED HU min**	93.50 (90.50 to 96.50)	96 (NC)	0.21
**DISCRETIZED HU mean**	107.74 (106.86 to 108.64)	109.16 (NC)	0.09
**DISCRETIZED HU std**	2.48 (2.31 to 2.79)	2.43 (NC)	0.46
**DISCRETIZED HU max**	117 (115.50 to 118.50)	115 (NC)	0.68
**DISCRETIZED HU Q1**	106 (105 to 107)	108 (NC)	**0.049**
**DISCRETIZED HU Q2**	108 (107 to 109)	109 (NC)	0.13
**DISCRETIZED HU Q3**	109 (109 to 110)	111 (NC)	0.053
**DISCRETIZED HU Skewness**	−0.43 (−0.59 to −0.18)	−0.68 (NC)	0.08
**DISCRETIZED HU Kurtosis**	3.85 (3.43 to 4.28)	4.49 (NC)	0.22
**DISCRETIZED HU ExcessKurtosis**	0.85 (0.43 to 1.28)	1.49 (NC)	0.22
**DISCRETIZED HISTO Entropy log10**	1 (0.97 to 1.05)	0.98 (NC)	0.41
**DISCRETIZED HISTO Entropy log2**	3.34 (3.21 to 3.48)	3.27 (NC)	0.41
**DISCRETIZED HISTO Energy [=Uniformity]**	0.12 (0.11 to 0.13)	0.13 (NC)	0.26
**DISCRETIZED AUC CSH**	1077 (1068 to 1086)	1091 (NC)	0.09
**GLCM Homogeneity [=InverseDifference]**	0.46 (0.44 to 0.47)	0.48 (NC)	0.54
**GLCM Energy [=AngularSecondMoment]**	0.017 (0.015 to 0.021)	0.022 (NC)	0.18
**GLCM Contrast [=Variance]**	6.64 (5.87 to 7.88)	5.75 (NC)	0.6
**GLCM Correlation**	0.38 (0.34 to 0.45)	0.30 (NC)	0.18
**GLCM Entropy log10**	1.92 (1.85 to 1.99)	1.83 (NC)	0.19
**GLCM Entropy log2 [=JointEntropy]**	6.39 (6.16 to 6.61)	6.07 (NC)	0.19
**GLCM Dissimilarity**	1.97 (1.84 to 2.15)	1.82 (NC)	0.57
**GLRLM SRE**	0.90 (0.89 to 0.91)	0.90 (NC)	0.63
**GLRLM LRE**	1.53 (1.47 to 1.59)	1.53 (NC)	0.73
**GLRLM LGRE**	8.6E-5 (8.5E-5 to 8.8E-5)	8.4E-5 (NC)	0.09
**GLRLM HGRE**	11605 (11418 to 11794)	11908 (NC)	0.09
**GLRLM SRLGE**	7.7E-5 (7.6E-5 to 7.9E-5)	7.7E-5 (NC)	0.24
**GLRLM SRHGE**	10462 (10251 to 10602)	10702 (NC)	0.13
**GLRLM LRLGE**	1.3E-4 (1.3E-4 to 1.4E-4)	1.3E-4 (NC)	0.38
**GLRLM LRHGE**	17742 (17266 to 18648)	18235 (NC)	0.73
**GLRLM GLNU**	889.69 (498.56 to 1660.21)	469.51 (NC)	0.15
**GLRLM RLNU**	5609 (3221 to 11596)	3289 (NC)	0.09
**GLRLM RP**	0.87 (0.86 to 0.88)	0.87 (NC)	0.66
**NGLDM Coarseness**	0.86 (0.86 to 0.88)	0.0015 (NC)	0.14
**NGLDM Contrast**	0.04 (0.03 to 0.04)	0.04 (NC)	0.97
**NGLDM Busyness**	0.39 (0.21 to 0.63)	0.17 (NC)	0.11
**GLZLM SZE**	0.64 (0.62 to 0.65)	0.64 (NC)	0.83

**GLZLM LZE**	3204 (1110 to 8153)	1226 (NC)	0.19
**GLZLM LGZE**	8.8E-5 (8.6E-5 to 8.9E-5)	8.6E-5 (NC)	0.32
**GLZLM HGZE**	11412 (11265 to 11640)	11684 (NC)	0.32
**GLZLM SZLGE**	5.6E-5 (5.4E-5 to 5.8E-5)	5.6E-5 (NC)	0.93
**GLZLM SZHGE**	7250 (7054 to 7406)	7434 (NC)	0.41
**GLZLM LZLGE**	0.27 (0.09 to 0.71)	0.10 (NC)	0.19
**GLZLM LZHGE**	3.79E+7 (1.30E+7 to 9.41E+7)	1.48E+7 (NC)	0.19
**GLZLM GLNU**	113.51 (67.20 to 191.10)	71.75 (NC)	0.13
**GLZLM ZLNU**	508.48 (320.11 to 928.6)	357.87 (NC)	0.16
**GLZLM ZP**	0.16 (0.13 to 0.19)	0.17 (NC)	0.22

Legend: **AUC:** area under the curve; **CSH**: cumulative SUV-volume histogram**; GLCM**: gray-level-co-occurrence-matrix; **GLNU**, gray-level nonuniformity; **GLRLM**, gray-level-run-length-matrix; **GLZLM**: gray-level zone-length-matrix**; HGRE:** high-gray-level-emphasis**; HGZE**: high-gray-level-zone-emphasis; **HU:** Hounsfield-Unit**; LGZE:** low-gray-level-zone-emphasis; **LRE:** long-run-emphasis; **LRHGE:** long-run-high-gray-level-emphasis; **LRLGE:** long-run-low-gray-level-emphasis; **LZE**: large-zone-emphasis**; LZHGE**: large-zone-high-gray-level-emphasis; **; LZLGE**: large-zone-low-gray-level-emphasis; **NGLDM**: neighboring-gray-level-dependence-matrix; **Q1-2-3**: quartile 1-2-3; **RLNU**: run-length-non-uniformity**; RP:** Run-percentage; **SRHGE**: short-run-high-gray-level-emphasis**; SRLGE**: short-run-low-gray-level-emphasis; **SZHGE**: small-zone-high-gray-level-emphasis; **SZLGE**: small-zone-low-gray-level-emphasis**; SRE**: short-run-emphasis; **Std:** standard deviation; **SZE**: small-zone-emphasis; **SZHGE**: short-zone-high-gray-level-emphasis; ; **SZLGE**: short-zone-low-gray-level emphasis; **YI**: Youden Index; **ZLNU:** zone-length-non-uniformity; **ZP**: zone-percentage.

Table S4. Microsatellite status: values of the 56 texture parameters. Four significant parameters were found.

Texture parameter	Median (interquartile range)		p
	MSS (group 0)	MSI (group 1)	
**CONVENTIONAL HU min**	−61.30 (−93.12 to −39.83)	−76.30 (−212.44 to −43.22)	0.21
**CONVENTIONAL HU mean**	74.97 (65 to 82.01)	65.11 (58.35 to 81.48)	0.2
**CONVENTIONAL HU std**	24.56 (22.19 to 26.54)	27.89 (23.37 to 30.25)	0.099
**CONVENTIONAL HU max**	159.52 (148.75 to 174.51)	169.04 (159.19 to 184.83)	0.27
**CONVENTIONAL HU Q1**	60.22 (48.68 to 68.48)	47.82 (38.92 to 63.58)	0.11
**CONVENTIONAL HU Q2**	76.19 (66.79 to 85.07)	66.23 (57.26 to 83.37)	0.17
**CONVENTIONAL HU Q3**	91.92 (82.76 to 99.95)	84 (76.46 to 100.78)	0.32
**CONVENTIONAL HU Skewness**	−0.45 (−0.63 to −0.22)	−0.44 (−0.72 to −0.21)	0.94
**CONVENTIONAL HU Kurtosis**	3.93 (3.44 to 4.56)	4.01 (3.57 to 4.73)	0.71
**CONVENTIONAL HU ExcessKurtosis**	0.93 (0.44 to 1.56)	1.01 (0.57 to 1.73)	0.71
**DISCRETIZED HU min**	94 (91 to 97)	93 (79 to 96)	0.18
**DISCRETIZED HU mean**	108 (107 to 108.7)	107.01 (106.33 to 108.65)	0.2
**DISCRETIZED HU std**	2.48 (2.23 to 2.67)	2.8 (2.36 to 3.04)	0.094
**DISCRETIZED HU max**	116 (115 to 118)	117 (116.5 to 119)	0.2
**DISCRETIZED HU Q1**	106.5 (105 to 107)	105 (104.75 to 107)	0.14
**DISCRETIZED HU Q2**	108 (107 to 109)	107 (106 to 109)	0.24
**DISCRETIZED HU Q3**	110 (109 to 110)	109 (108 to 110.25)	0.17
**DISCRETIZED HU Skewness**	−0.44 (−0.62 to −0.21)	−0.44 (−0.7 to −0.21)	0.91
**DISCRETIZED HU Kurtosis**	3.88 (3.44 to 4.49)	3.98 (3.56 to 4.72)	0.71
**DISCRETIZED HU ExcessKurtosis**	0.88 (0.44 to 1.49)	0.98 (0.56 to 1.72)	0.71
**DISCRETIZED HISTO Entropy log10**	1 (0.96 to 1.03)	1.06 (0.97 to 1.09)	0.079
**DISCRETIZED HISTO Entropy log2**	3.33 (3.2 to 3.41)	3.51 (3.23 to 3.63)	0.079
**DISCRETIZED HISTO Energy [=Uniformity]**	0.12 (0.11 to 0.13)	0.1 (0.093 to 0.13)	0.074
**DISCRETIZED AUC CSH**	1079 (1070 to 1087)	1070 (1063 to 1086)	0.2
**GLCM Homogeneity [=InverseDifference]**	0.46 (0.44 to 0.48)	0.45 (0.43 to 0.48)	0.73
**GLCM Energy [=AngularSecondMoment]**	0.018 (0.015 to 0.021)	0.013 (0.012 to 0.02)	0.084
**GLCM Contrast [=Variance]**	6.59 (5.75 to 7.73)	7.59 (5.74 to 8.9)	0.52
**GLCM Correlation**	0.37 (0.34 to 0.44)	0.43 (0.38 to 0.52)	0.089
**GLCM Entropy log10**	1.91 (1.85 to 1.96)	2.04 (1.85 to 2.06)	0.099
**GLCM Entropy log2 [=JointEntropy]**	6.35 (6.16 to 6.5)	6.76 (6.15 to 6.86)	0.099
**GLCM Dissimilarity**	1.95 (1.82 to 2.13)	2.13 (1.82 to 2.23)	0.67
**GLRLM SRE**	0.9 (0.89 to 0.91)	0.89 (0.88 to 0.91)	0.53
**GLRLM LRE**	1.53 (1.46 to 1.56)	1.55 (1.44 to 1.67)	0.55
**GLRLM LGRE**	8.6E-5 (8.5E-5 to 8.8E-5)	8.8E-5 (8.5E-5 to 8.9E-5)	0.21
**GLRLM HGRE**	11661 (11448 to 11815)	11452 (11309 to 11804)	0.21
**GLRLM SRLGE**	7.7E-5 (7.6E-5 to 7.9E-5)	7.7E-5 (7.7E-5 to 7.9E-5)	0.77
**GLRLM SRHGE**	10475 (10263 to 10604)	10318 (10115 to 10655)	0.37
**GLRLM LRLGE**	1.3E-4 (1.3E-4 to 1.4E-4)	1.4E-4 (1.2E-4 to 1.4E-4)	0.47
**GLRLM LRHGE**	17739 (17197 to 18235)	17830 (16943 to 19052)	0.61
**GLRLM GLNU**	655 (469 to 1468)	1448 (772 to 3213)	0.084
**GLRLM RLNU**	4419 (2811 to 9267)	11829 (5918 to 21721)	**0.037**
**GLRLM RP**	0.87 (0.86 to 0.88)	0.86 (0.84 to 0.88)	0.57
**NGLDM Coarseness**	1.2E-3 (5.4E-4 to 1.7E-3)	4.9E-4 (2.8E-4 to 9.3E-4)	0.055
**NGLDM Contrast**	0.036 (0.028 to 0.043)	0.03 (0.021 to 0.047)	0.52
**NGLDM Busyness**	0.34 (0.19 to 0.49)	0.63 (0.36 to 0.88)	0.066
**GLZLM SZE**	0.64 (0.63 to 0.65)	0.62 (0.62 to 0.63)	0.062
**GLZLM LZE**	2407 (1086 to 6361)	4790 (1953 to 18584)	0.27
**GLZLM LGZE**	8.8E-5 (8.6E-5 to 8.9E-5)	8.9E-5 (8.6E-5 to 8.9E-5)	0.42

**GLZLM HGZE**	11458 (11275 to 11668)	11355 (11219 to 11639)	0.45
**GLZLM SZLGE**	5.6E-5 (5.5E-5 to 5.8E-5)	5.5E-5 (5.4E-5 to 5.6E-5)	0.23
**GLZLM SZHGE**	7334 (7114 to 7457)	7070 (6937 to 7192)	**0.0081**
**GLZLM LZLGE**	0.2 (0.09 to 0.54)	0.42 (0.16 to 1.64)	0.27
**GLZLM LZHGE**	2.86E+7 (1.30E+7 to 7.59E+7)	5.41E+7 (2.32E+7 to 2.11E+7)	0.28
**GLZLM GLNU**	97.39 (62.29 to 177.79)	186.42 (133.31 to 290.23)	**0.025**
**GLZLM ZLNU**	378 (304 to 763)	920 (546 to 1378)	**0.011**
**GLZLM ZP**	0.16 (0.14 to 0.19)	0.15 (0.12 to 0.2)	0.3

Legend: **AUC:** area under the curve; **CSH**: cumulative SUV-volume histogram**; GLCM**: gray-level-co-occurrence-matrix; **GLNU**, gray-level nonuniformity; **GLRLM**, gray-level-run-length-matrix; **GLZLM**: gray-level zone-length-matrix**; HGRE:** high-gray-level-emphasis**; HGZE**: high-gray-level-zone-emphasis; **HU:** Hounsfield-Unit**; LGZE:** low-gray-level-zone-emphasis; **LRE:** long-run-emphasis; **LRHGE:** long-run-high-gray-level-emphasis; **LRLGE:** long-run-low-gray-level-emphasis; **LZE**: large-zone-emphasis**; LZHGE**: large-zone-high-gray-level-emphasis; **; LZLGE**: large-zone-low-gray-level-emphasis; **NGLDM**: neighboring-gray-level-dependence-matrix; **Q1-2-3**: quartile 1-2-3; **RLNU**: run-length-non-uniformity**; RP:** Run-percentage; **SRHGE**: short-run-high-gray-level-emphasis**; SRLGE**: short-run-low-gray-level-emphasis; **SZHGE**: small-zone-high-gray-level-emphasis; **SZLGE**: small-zone-low-gray-level-emphasis**; SRE**: short-run-emphasis; **Std:** standard deviation; **SZE**: small-zone-emphasis; **SZHGE**: short-zone-high-gray-level-emphasis; ; **SZLGE**: short-zone-low-gray-level emphasis; **YI**: Youden Index; **ZLNU:** zone-length-non-uniformity; **ZP**: zone-percentage.
